# A Retrospective Study of Survivors of Endovascular Coiling for Posterior and Anterior Aneurysms: Medical and Patient Perspectives

**DOI:** 10.1097/MD.0000000000001313

**Published:** 2015-08-14

**Authors:** Sarah J. Wilson, Ruth Drackford, Michael Holt

**Affiliations:** From the Melbourne School of Psychological Sciences (SJW, RD), The University of Melbourne, Parkville; and Department of Radiology (MH), Monash Medical Centre, Clayton, Victoria, Australia.

## Abstract

This article documents the longer-term medical and psychosocial outcomes of patients referred for endovascular coiling.

There is limited research investigating outcome following endovascular coiling for posterior compared to anterior circulation aneurysms, and minimal understanding of how medical outcomes relate to patient experiences of treatment and quality of life.

We studied a consecutive cohort of 80 patients referred Australia wide for endovascular coiling between 1995 and 2003 (49% posterior; 76% ruptured; 69% women, mean age 51.5 years). We used a mixed methods approach, assessing medical outcome with the Modified Rankin Scale (MRS) in 61 patients (76%), and health-related quality of life and psychosocial functioning using the EuroQol questionnaire and a qualitative semistructured interview in 49 patients (61%).

Despite the high proportion of posterior aneurysms, the majority of patients (80%) showed good medical outcomes as indicated by regained independence of activities of daily living (MRS score ≤3). Patients with unruptured aneurysms were significantly more likely to show good outcomes (*P* < 0.04), whereas aneurysm location (posterior, anterior, or mixed) showed no significant effect. In patients with good medical outcomes, greater functional disability was associated with neurological complications surrounding treatment (*P* < 0.05). Good outcomes correlated with higher EuroQol ratings (*P* < 0.001) and the experience of less change after treatment (*P* < 0.001), although psychosocial adjustment issues were reported by most of the patients, including those with no medical symptoms.

These results support the long-term efficacy of endovascular coiling, particularly for posterior circulation aneurysms. They have implications for guiding clinicians and patients in their choice of treatment, as well as the provision of psychological counseling for patient adjustment issues posttreatment.

## INTRODUCTION

Aneurysm prevalence rates vary in the literature. Early autopsy studies suggested an incidence rate of between 2% and 5%^1,2^; however, a more recent angiographic review indicated a prevalence rate of only 0.65%.^3^ As 80% to 85% of aneurysms occur in the anterior circulation,^4^ it is not surprising that much of the literature has focused on the outcomes of these aneurysms.^5–8^ It is estimated that in Western populations, aneurysmal subarachnoid hemorrhage (SAH) occurs in 6 to 8 people/100,000.^9^ The population-based fatality rate following neurosurgical treatment for aneurysmal SAH remains high, and up to 80% of patients show decreased health-related quality of life and cognitive impairments 1 year after SAH.^10–12^

Although neurosurgical clipping has been the traditional treatment for ruptured aneurysms, endovascular coiling has been recently developed as a less-invasive alternative.^13^ The International Subarachnoid Aneurysm Trial (ISAT)^7^ compared the effects of these 2 techniques on general patient functioning, as assessed by the Modified Rankin Scale (MRS).^14^ Poorer outcomes were documented for the neurosurgical group, with the endovascular coiling group showing a reduced relative risk of death or significant disability at 1 year posttreatment. Recent clinical outcome data for the same cohort at 5 years showed similar findings.^8^

The ISAT sample composed of mostly anterior aneurysms (97%), with the results demonstrating the clear benefit of endovascular coiling for this population.^15^ The present study boasts a high proportion of posterior circulation aneurysms, the medical outcome of which tends to be worse following neurosurgical clipping.^16^ The medical outcome of posterior aneurysms following endovascular coiling remains largely unexplored, providing the impetus for the current study.

Also novel to this study, previous research has focused on medical outcome, with less effort to contextualize this outcome within the subjective experience of the patient. Can we comfortably predict that a good medical outcome means the patient ultimately experiences a good psychosocial recovery and posttreatment experience? Evidence suggests that crude outcome scales can “mask” the real issues for patients, underpinning our second aim to more fully characterize these issues in our sample using an in-depth, qualitative approach.^17^

Third, the present study sought one of the longest follow-ups to date, which is important given the relative newness of the endovascular coiling procedure. We focused on the location (anterior, posterior, or mixed) and presentation (ruptured or unruptured) of patient aneurysms as key variables affecting medical and psychosocial outcomes, and explored the relationship between subjective patient experiences of life after treatment and medical outcomes. Given limited investigation of posterior aneurysms to date, we used a mixed qualitative and quantitative approach to fully document the nature and range of issues experienced by patients.

## MATERIALS AND METHODS

### Participants

A consecutive cohort of 80 patients underwent endovascular coiling for intracranial aneurysms between 1995 and 2003, with referrals accepted from medical professionals Australia wide. All procedures were performed by 2 neuroradiologists. On clinical presentation, 61 of the 80 patients (76%) had ruptured aneurysms and 19 (24%) had unruptured aneurysms. Twenty-nine patients (36%) had aneurysms in the anterior circulation, 39 (49%) in the posterior circulation, and 12 (15%) had multiple aneurysms involving both locations. Forty-four patients (55%) experienced neurological complications, including hydrocephalus before or during coiling, perioperative vasospasm, use of external ventricular drainage, and perioperative or postoperative stroke (not SAH). Complication rates were similar for posterior aneurysms (54%), anterior aneurysms (55%), and patients with aneurysms in both the locations (58%). The mean age of the patient cohort was 51.5 years (range 25–80 years), and 55 (69%) were women. Each patient received routine medical review posttreatment, with the length of clinical follow-up specific to the patient's rate of recovery. All patients were routinely prescribed Aspirin 100 mg daily for 3 to 6 months postcoiling, after which time this treatment was optional. No other medications were routinely prescribed.

### Procedure and Measures

Medical outcome was assessed using the MRS, which determines the level of functional capacity and independence of the patient.^14^ Quantitative assessment of the patient's perceived ‘health status’ after treatment was obtained with the EuroQol, which is a previously published measure of health-related quality of life with acceptable reliability and validity.^18,19^ Qualitative descriptions of each patient's subjective experience of treatment outcome were obtained using a purpose-developed semistructured psychosocial interview. This assessed the patient's experience of medical treatment and its perceived effects on life posttreatment by canvassing 8 domains identified as important in the literature.^20–24^ These included ongoing symptoms, functional change, level of dependency, cognitive change, emotional change, social change, change in personality, and psychological change. For each domain, patients were asked open-ended questions followed by specific probes, using premorbid functional abilities, to contextualize the extent of any change posttreatment. The interviews were audio recorded and patient responses transcribed verbatim. Content analysis was used to characterize commonly occurring themes reported by patients and to construct a profile of psychosocial change for each patient pretreatment to posttreatment. Ten percent of the interviews were independently coded by a researcher who was blind to patient's medical status. This showed a high level of interrater agreement (100%) for the presence or absence of change in each of the 8 domains.

Substantive attempts were made to track all patients in the cohort, first using most recent contact details, then through next of kin, the Australian electoral role, and the Death Registry of Victoria. Using this procedure, 69 of the 80 patients (86%) were located, with the remaining 11 (14%) lost to follow-up after discharge from outpatients. Located patients were offered the choice to participate in a follow-up assessment in their own homes or at The University of Melbourne, Parkville, Australia. The assessment took approximately 2 hours and included administration of the MRS, the EuroQol, and the psychosocial interview. A subgroup of patients also underwent detailed neuropsychological assessment, which is reported elsewhere.^25^ The study was conducted in accordance with the institutional guidelines and approved by the relevant Human Research Ethics Committees. All participants gave written informed consent, and none were compensated for participating in the study.

### Data Analyses

All statistical analyses were performed using IBM SPSS Statistics (version 19.0.0 for Mac OS X), with 2-tailed *P* ≤ 0.05 used to determine statistical significance. To assess the impact of aneurysm location (anterior, posterior, or mixed) and presentation (ruptured or unruptured) on medical outcomes, we first used χ^2^ tests to compare patients who had survived and regained independence of activities of daily living (good medical outcomes, MRS ≤3) to those who had not survived or were suffering from moderately severe disability (poor medical outcomes, MRS ≥4). Similarly, we used χ^2^ analysis to examine any effects due to neurological complications at the time of the endovascular coiling procedure.

Only those patients who had survived and regained independence of activities of daily living were able to complete the detailed qualitative and quantitative psychosocial outcome assessment. This allowed us to examine reports of any ongoing cognitive or psychosocial difficulties in patients with good medical outcomes.^12^ In particular, we used a 2 × 3 factor analysis of variance (ANOVA) to further examine the influence of aneurysm location, presentation, and the presence of neurological complications on patient MRS scores (range 0–3). We also used a 2 × 3 factor ANOVA for our quantitative psychosocial outcome assessment, examining the effects of aneurysm location, presentation, and neurological complications on total EuroQol health status scores. Qualitatively, we profiled patient experiences of psychosocial changes pretreatment to posttreatment based on common themes identified from content analysis of the psychosocial interviews. Using a 2 × 3 factor ANOVA, we then examined the proportion of patients reporting psychosocial changes as a function of aneurysm location, presentation, and neurological complications.

Finally, we assessed the relationship between each patient's subjective experience of treatment outcome and objective medical outcome in 2 ways. First, by correlating each patient's total EuroQol health status score with the MRS score (range 0–3), and second by correlating the proportion of patients reporting psychosocial changes posttreatment with MRS scores.

## RESULTS

### Medical Outcomes

Of the 69 located patients, 7 (10%) were deceased. The cause of death in 2 was an aneurysmal SAH, whereas a third patient suffered a SAH but it was unknown whether this was aneurysmal. In 2 patients, the cause of death was unrelated (metastatic melanoma, and pneumonia with complications), and unknown for the remaining 2 patients. Five of the 69 patients (7%) were too unwell to participate in the psychosocial assessment, with 2 of these likely the result of poor aneurysm outcome. The remaining 3 reflected individual circumstances, such as cancer and age-related frailty. Eight patients (12%) declined participation, with reasons not given by the majority of patients. This resulted in a sample of 61 patients for whom MRS scores were assigned (Table 1), including the 5 unwell patients who were broadly classified to categories 4 and 5 (collapsed), reflecting moderately severe to severe disability posttreatment.

**TABLE 1:**
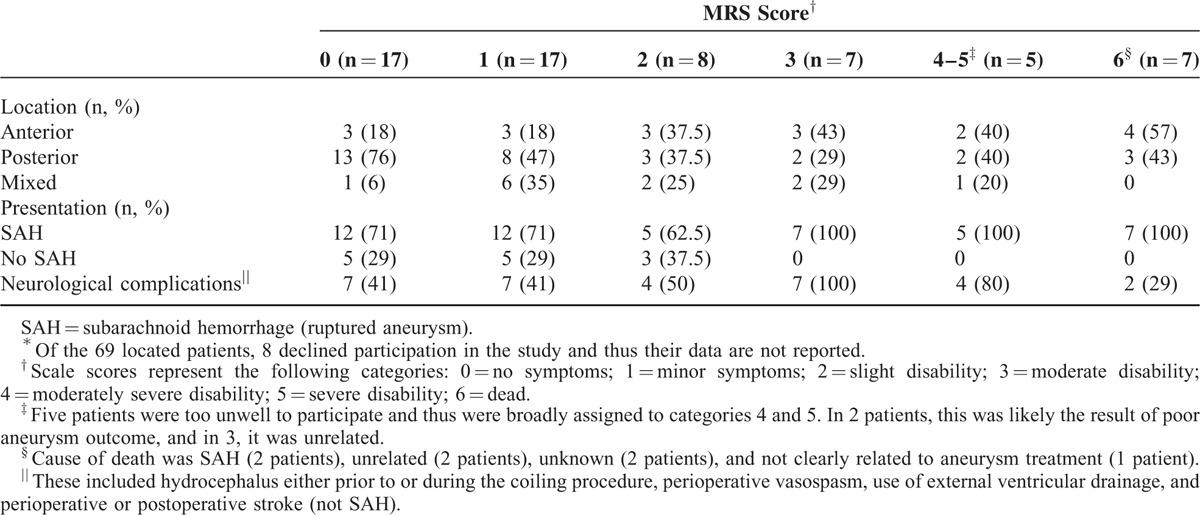
Modified Rankin Scale (MRS) Scores Shown for Aneurysm Location and Presentation (N = 61^∗^)

Of the 61 patients, the majority (n = 49, 80%) had good medical outcomes with MRS scores falling in the range of 0 to 3 (no symptoms to moderate disability). The remaining 12 patients (20%) had poor medical outcomes with MRS scores of 4 to 6 (moderately severe disability to dead). Chi-squared analyses indicated that the location of the aneurysm (anterior, posterior, or mixed) was not associated with good or poor medical outcomes (*P* > 0.05), whereas the presentation of the aneurysm (ruptured or unruptured) had a significant impact (χ^2^_(1)_ = 4.05, *P* < 0.05), with all 12 patients with poor medical outcomes having ruptured aneurysms (100%). The presence of neurological complications at the time of endovascular coiling showed no significant effect (*P* > 0.05; Table 1).

On average, the 49 patients with good medical outcomes were 5.6 years posttreatment (range 1–11 years) at the time of participation. They had a mean age of 50.9 years (range 25–76 years) and 34 (69%) were women, similar to the larger cohort. A 2 × 3 ANOVA showed a significant main effect of neurological complications (F_(1,48)_ = 4.17, *P* < 0.05, partial η^2^ = 0.10), with the group with neurological complications scoring higher on the MRS [mean (M) = 1.44, standard deviation (SD) = 1.19] than the group without neurological complications (M = 0.75, SD = 0.74). There were no main effects of aneurysm location or presentation on MRS scores, and no significant 2 or 3-way interactions between aneurysm location, presentation, or neurological complications (*P* > 0.05 for all comparisons). These findings indicate a higher level of disability in patients with good medical outcomes if they experienced a neurological complication at the time of treatment, independent of aneurysm location or presentation.

### Psychosocial Outcomes: Patient Quality of Life

Overall, the total EuroQol scores of the 49 patients with good medical outcomes indicated a high positive mean rating of health status (M = 0.80, SD = 0.21, maximum score 1.0). This represents “full health,” that is, no difficulties in any of the 5 domains canvassed by the EuroQol, including mobility, personal care, usual activities, pain/discomfort, and anxiety/depression.^19^ The mean subjective rating of quality of life (made on a visual analog scale) was also high (M = 78.3, SD = 15.9, maximum score 100), indicating that patients generally reported a “good health state.” A 2 × 3 factor ANOVA showed no significant interactions or main effects for the location or presentation of the aneurysm, or the presence of neurological complications on EuroQol health status scores (*P* > 0.05 for all comparisons). Similarly, there were no significant interactions or main effects for the visual analog scale (*P* > 0.05 for all comparisons). Finally, Spearman rank correlation coefficient showed that patient reports of psychosocial disability on the EuroQoL correlated strongly with their level of medical disability recorded on the MRS (Spearman *ρ* = −0.566, *P* < 0.001).

### Psychosocial Outcomes: Patient Experiences of Medical Outcome

The majority of patients undergoing detailed qualitative assessment reported change in at least one area of their life posttreatment (n = 39/49, 80%), as indicated by a positive response to 1 of the 8 domains canvassed by the psychosocial interview. Table 2 shows the frequency of reports of change for each domain accompanied by individual patient quotes that capture the nature of patient experiences. These quotes were considered in light of the entire interview to ensure they were not taken out of context and to maintain the patient's original perspective. They illustrate different themes of change that were common in the data and provide examples of how different issues manifested for patients.

**TABLE 2:**
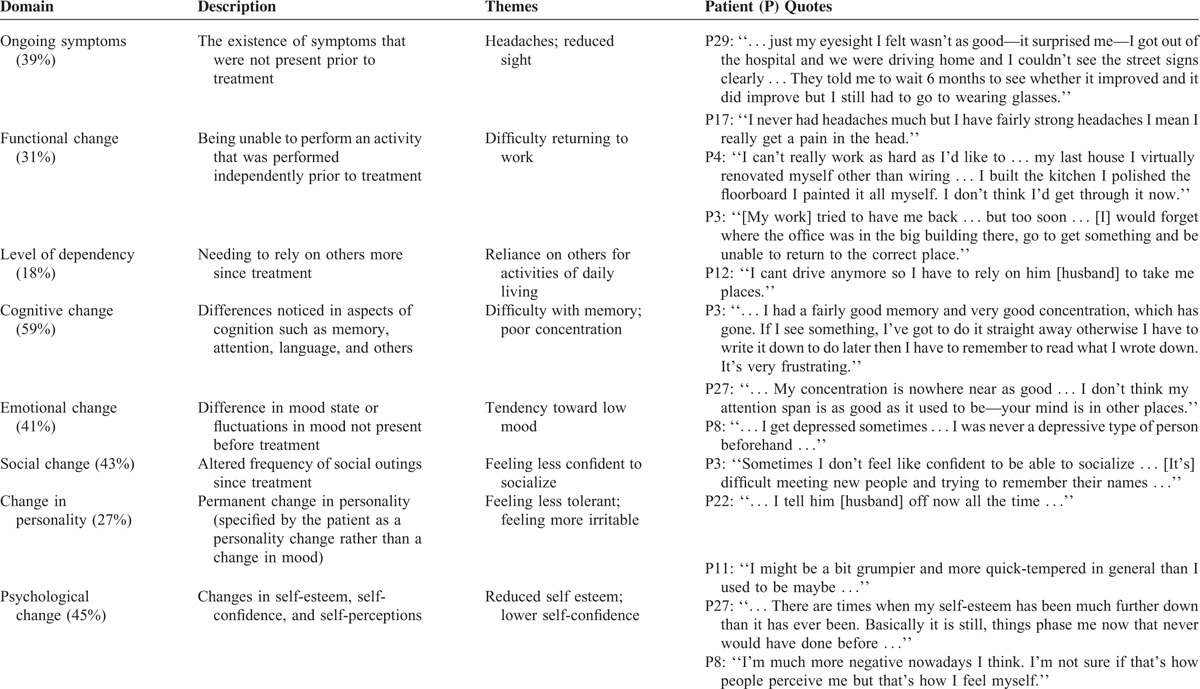
Characterization of Key Issues Identified by Patients and Their Frequency of Report (%)

Cognitive changes were reported most frequently (59%), followed by changes in psychological (45%), social (43%), and emotional (41%) functioning. Cognitive changes manifested in different ways, with poorer memory and concentration most commonly reported. Patients described these changes in a pragmatic sense, for example, needing to be reminded of the content of previous conversations and feeling easily distracted or unable to focus on specific tasks. For emotional functioning, downward changes in mood predominated, with increased frequency of low mood commonly identified. Many patients who felt this noted that it represented a significant drop from their previous sense of emotional well-being. Some patients were able to pinpoint the “reactive” nature of episodes of low mood, for example, difficulty performing well at work. Others felt they were more prone to become “depressed” at a general level posttreatment.

The psychological issues raised by patients primarily reflected changes in self-esteem and self-confidence. For some patients, their self-perceptions had altered from viewing themselves as competent and able to handle a multitude of situations, to less capable and more easily “phased” by previously manageable situations. There was also a sense of increased vulnerability, with one patient referring to the frail and unpredictable nature of life. There was, however, an overall sense of acceptance of an adjustment process posttreatment—its ups and downs, and a strong feeling of moving forwards in life. Despite perceived change from a social perspective, many patients were able to return to their preoperative life roles at the same level of independence. The return to work rate was also high (77%) suggesting that reintegration into the workplace, and balancing the demands of this at a cognitive and emotional level, was successful for a substantial proportion of patients.

**FIGURE 1:**
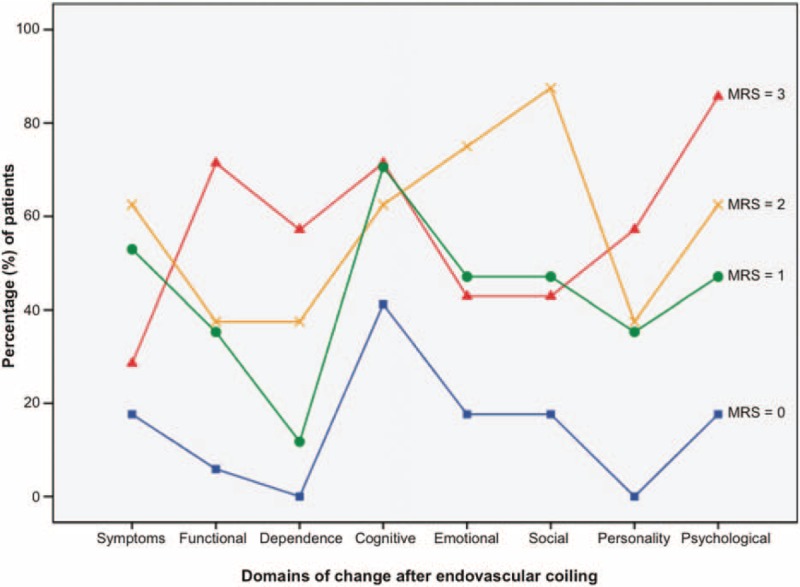
Percentage of patients who reported changes during the psychosocial interview (n = 49) following endovascular coiling shown relative to Modified Rankin Scale (MRS) scores, with blue square = no symptoms (score = 0), green circle = minor symptoms (score = 1), orange cross = slight disability (score = 2), and red triangle = moderate disability (score = 3). The 8 domains of change canvassed during the interview included ongoing symptoms, functional change, level of dependency, cognitive change, emotional change, social change, change in personality, and psychological change (see Table 2 for definitions and examples).

Figure 1 demonstrates the percentage of patients reporting psychosocial changes in each of the 8 domains relative to their MRS scores. It shows that patients with higher scores (slightly moderate disability) were generally more likely to report changes in their life after treatment, with cognitive, psychological, and social changes again prominent. Patients with moderate disability (MRS score =3) were also more likely to report a change in their ability to perform work activities at a level commensurate with their pretreatment performance. In contrast, patients with the best medical outcomes (MRS score =0) had a profile of the least change. Interestingly, patients with minor symptoms (MRS score =1) showed a similar profile, albeit with a somewhat higher report of change.

The Spearman rank correlation coefficient for MRS scores and the proportion of changes reported by patients across the 8 domains showed that posttreatment changes strongly correlated with the patient's level of disability recorded on the MRS (Spearman *ρ* = 0.553, *P* < 0.001). In contrast, a 2 × 3 factor ANOVA showed no significant interactions or main effects for the location or presentation of the aneurysm, or the occurrence of neurological complications on the proportion of changes reported across the 8 domains (*P* > 0.05 for all comparisons). This suggests no significant influence of these variables on the experience of change described by patients with good medical outcomes after treatment.

## DISCUSSION

This study employed a mixed qualitative and quantitative approach to examine longer-term medical and psychosocial outcomes of survivors of endovascular coiling particularly in the posterior circulation. Four key findings were observed. First, ruptured aneurysms were associated with poorer medical outcomes, as characterized by higher mortality rates and reduced independence of daily living. This confirms the results of the previous research focusing on aneurysms in the anterior circulation,^10–12^ and extends the findings to a cohort of patients with a majority of posterior circulation aneurysms. Second, no differences in medical outcomes were observed for aneurysm location, providing important new data consistent with the idea that endovascular coiling fares equally well in patients with anterior, posterior, or mixed locations. Third, the occurrence of neurological complications was the only factor to differentiate greater functional disability in patients with good medical outcomes, whereas aneurysm location and presentation showed no such effects. Fourth, there was a strong mapping between medical outcomes, quantitative measures of health-related quality of life, and patient experiences of change after treatment, with greater functional change and lower quality of life associated with poorer medical outcomes. However, despite good medical outcomes in the majority of patients (80%), most reported psychosocial changes after treatment, including those with no symptoms (MRS score of 0). This implies that all patients may benefit from psychological support as they adjust to these changes after treatment, irrespective of their medical outcomes.

Importantly, this study adds new data demonstrating positive medical and psychosocial outcomes in patients with posterior circulation aneurysms after endovascular coiling. Previous research on the use of endovascular coiling for posterior circulation aneurysms has demonstrated its effectiveness primarily in terms of reduced mortality and morbidity.^26–28^ The present study extends this work by systematically investigating psychosocial functioning posttreatment using both quantitative and qualitative approaches. It indicates that the location of the aneurysm (anterior, posterior, or mixed) did not have significant effects, either on medical outcomes or perceived changes in psychosocial functioning. This contrasts with poorer outcomes previously shown for patients undergoing neurosurgical clipping of posterior as compared to anterior circulation aneurysms,^16^ highlighting the effectiveness of endovascular coiling as an alternative treatment, particularly for posterior circulation aneurysms.

The most common psychosocial changes perceived by patients after endovascular coiling included altered cognitive, psychological, emotional, and social functioning. This was typically characterized by subjective reports of poor memory and concentration, increased symptoms of depression, and lowered self-confidence, often resulting in reduced social activity. These perceived changes were evident even in patients with the best medical outcomes, pointing to a process of psychological and social adjustment experienced by the majority of patients after treatment. In other words, good outcomes measured by quantitative medical scales may mask the reality of the experience of the patient. This is a key finding which has not been previously documented in the literature. Reassuringly, although the results suggest that the adjustment process is characterized by significant psychosocial change, the return to work rate was high indicating that most patients were able to reintegrate into the workplace.

Previous research investigating psychosocial outcomes following neurosurgical clipping has shown that a significant proportion of patients report changes in personality and difficulties with their memory 4 to 7 years posttreatment.^21^ In the present study, which includes patients up to 11 years postcoiling, the same kinds of difficulties were described, including disturbance in mood, increased dependence, and changes in social functioning.^23^ This suggests that these types of changes may not be specific to neurosurgical treatment, but rather form part of a general process of psychological adjustment following treatment for intracranial aneurysms. Given debate about the early benefits of coiling over clipping and the extent to which these benefits remain at longer-term follow-up,^15,29,30^ it is conceivable that the timing of psychosocial changes may differ between treatments; however, this warrants investigation.

The majority of patients in our cohort had ruptured aneurysms (76%), limiting exploration of the effects of aneurysm presentation on patient outcomes. Nonetheless, combined with previous research, the findings indicate that aneurysmal SAH constitutes a key factor affecting outcome.^31,32^ In our study, all patients (100%) with poor medical outcomes (MRS scores of 4–6) had ruptured aneurysms. There is a paucity of research investigating the effects of neurological complications on psychosocial functioning after endovascular coiling; however, the neurosurgical literature suggests that these can have significant effects on the patient's level of long-term disability and psychosocial functioning.^21^ In the present study, there was a similar proportion of patients with and without neurological complications (∼50%), and their occurrence was associated with significantly greater functional disability in patients with good medical outcomes. This provides new evidence for the relevance of neurological complications as a predictor of outcome, regardless of the type of intervention.

The limitations of this study primarily relate to its cross-sectional design and its relatively small sample size, due to loss of patients to follow-up or their inability to participate in detailed psychosocial assessment because of poor medical outcomes. These limitations may, in part, reflect the number of patients with ruptured aneurysms in our cohort. However, despite the high population-based fatality rate of aneurysmal SAH, this study found that survivors of endovascular coiling, particularly in the posterior circulation, generally show good medical outcomes and positive ratings of health-related quality of life as they adjust to a range of psychosocial changes following treatment.

## CONCLUSIONS

Our findings contribute important new information to the underresearched area of longer-term outcomes of endovascular coiling for posterior circulation aneurysms. These findings have significant clinical implications, providing prognostic information for clinicians and patients to aid their ability to make informed decisions about treatment. The clinician can also counsel patients about psychosocial adjustment issues commonly experienced following treatment, including subjective changes in cognition, and altered psychological, emotional, and social functioning. The important finding that patients can experience psychosocial adjustment difficulties after treatment, even in the absence of medical symptoms, highlights that all patients may benefit from routine psychological support, irrespective of their medical outcomes. Normalizing these experiences can aid patient recovery by making the process of adjustment less fearful and unpredictable. The high return to work rates may also serve to reassure patients of a positive onward pathway over the longer term.

## ACKNOWLEDGMENTS

The authors would sincerely like to thank the patients for voluntarily giving up their time, without compensation, in order to participate in the study, including the lengthy distance some patients chose to travel. The authors would also like to acknowledge the assistance of the staff at Monash Medical Centre, particularly, the Neuroradiology Team, and Genevieve Rayner, for her assistance with manuscript preparation.
